# Enhancing nursing education to bolster nurse governance: insights from nurse managers

**DOI:** 10.3389/fmed.2023.1254428

**Published:** 2023-09-05

**Authors:** Sujin Choi

**Affiliations:** Department of Nursing, College of Medicine, Soonchunhyang University, Asan, Republic of Korea

**Keywords:** education, governance, communication, humanity, career, politics

## Abstract

**Aim:**

In South Korea, the level of nursing governance is moving toward shared governance. This study sought to explore nursing education contents in undergraduate nursing programs necessary to improve the governance of nurses from the perspectives of nurse managers.

**Methods:**

The study employs thematic analysis following the guidelines outlined in the Consolidated Criteria for Reporting Qualitative Research utilizing a qualitative research design. Our investigation involves general or tertiary hospital nurse managers intending to gain valuable insights and perspectives.

**Results:**

Interview data from 14 nurse managers were analyzed. A total of one main and four sub-themes were derived from the qualitative data analysis. Analysis revealed the main theme, “occupational socialization.” The four sub-themes were education on “nurse–patient and nurse-colleague communicative interaction,” “humanity,” “career development,” and “nurses as politicians.”

**Conclusion:**

The findings are valuable in suggesting critically needed educational content in undergraduate nursing programs to improve nursing governance. Future research should investigate the effects of the abovementioned themes on nursing governance among clinical nurses or nursing students for several years of follow-up data collection.

## 1. Introduction

Governance is a multidimensional concept that encompasses the structures and processes through which participants within an organization guide, manage, and oversee the goal-oriented endeavors of other members. (1). Hess (1) developed a measurement of governance that assessed six subdomains: control over nursing personnel, participation in committees, access to information, conflicts and goal settings, influence over resources, and control over nursing practice. Governance is categorized as traditional, shared, or self-governance depending on how much nurses have authority, access, opportunities, and participation in these subdomains. The critical concept of shared governance is shared decision-making related to the six subdomains between the nurses and nurse leaders (2). Shared governance promotes positive outcomes for patients and nurses, such as job satisfaction, turnover intention, and empowerment (3, 4).

However, a study on nurses working in tertiary general and general hospitals (5) in South Korea reported that they recognized the governance level as traditional. Another study on nurse managers (6) found that they recognized the level of nursing governance as shared governance, but at the initial level, which means that most of the authority and opportunity for decision-making lie with nurse managers. In traditional governance, nurses do not have authority, access, opportunities, or participation in the six subdomains, which may cause lower job satisfaction and turnover. According to a report by the Hospital Nurses Association (7), the nurse turnover rate is 14.5%, and the annual turnover rate for nurses with less than 1 year of experience was 34.1% in 2021. New nurses’ high turnover and low satisfaction rates affect healthcare organizations and nursing education institutions. The nursing education institutions provide nursing education and yield newly graduated nurses annually; efforts to improve nursing governance should be shared and supported.

Various studies in other countries regarding governance in nursing focused on the effect of shared governance of clinical nurses on nurse-side outcome variables such as satisfaction, nurse-sensitive indicators, and nurse engagement (8, 9), an integrative review of governance-strengthening strategies (10), governance of nursing students (11), and governance of nursing faculties (2, 12). However, in South Korea, except for studies on the current status of Korean nurses’ governance and its correlation with related variables in clinical settings (5, 6, 13, 14), nursing education research on the current state of governance or investigation of educational demand from clinical practice to improve governance in nursing has not been conducted. Therefore, research that can provide primary data to prepare educational content related to nursing governance is required.

Nurse managers are at the management level in healthcare organizations. They utilize the hospital’s policies, objectives, and plans for specific situations, reflect them in nursing practice, and direct, coordinate, and evaluate the clinical nursing activities performed by the nursing staff in the wards (15). Based on their critical roles in healthcare organizations, they can be equipped with an understanding of governance in nursing. A study on nurse managers’ perception of governance in nursing highlighted that nurses lack the perception of given authority (6). Korean nurses may have a relatively limited understanding of professional governance compared to nurse managers. Thus, this study explored nursing education content in undergraduate nursing programs necessary to improve the governance of nurses from the perspectives of nurse managers.

## 2. Methods

### 2.1. Design

A qualitative study design was chosen to explore how nurse managers perceive the necessary education in undergraduate nursing programs to improve nursing governance. The study’s reporting adhered to the Consolidated Criteria for Reporting Qualitative Research checklist or guideline.

### 2.2. Sampling strategy

The participants were nurse managers working in general and tertiary hospitals in the Seoul and Gyeonggi-do regions who voluntarily consented to participate. The inclusion criteria were at least 1 year of working experience as a nurse manager in a general or tertiary hospital. The author asked nurse executives in target hospitals to advertise this study to nurse managers; those interested provided their contact details voluntarily. The purpose and process of this study were explained to the participants. Participants were recruited until data saturation. The number of participants fulfills the required sample size for collecting qualitative data (16).

### 2.3. Data collection

Data were collected from May to June 2020 through individual interviews conducted by the author via telephone, given the COVID-19 restrictions. The author had experience conducting qualitative research on governance and other nursing-related topics. The interview time and date were set according to the participants’ preferences and convenience. The interview questions derived from the literatures (1, 5, 6, 10, 11) were: “What kind of education is necessary for undergraduate nursing programs to enhance control over nursing personnel, participation in committees, access to information, conflicts and goal settings, influence over resources, and control over nursing practice?” “Why do you think the kind of education should be needed?” “Could you tell me any episodes regarding nursing governance?” All interviews were audio-recorded, pseudonymized, and transcribed verbatim by the author. Recordings were deleted after data analysis was completed. Each interview lasted 35 min on average. The author wrote notes in the field immediately after each interview. Data analysis was performed concurrently with data collection.

### 2.4. Data analysis

**Figure 1:**
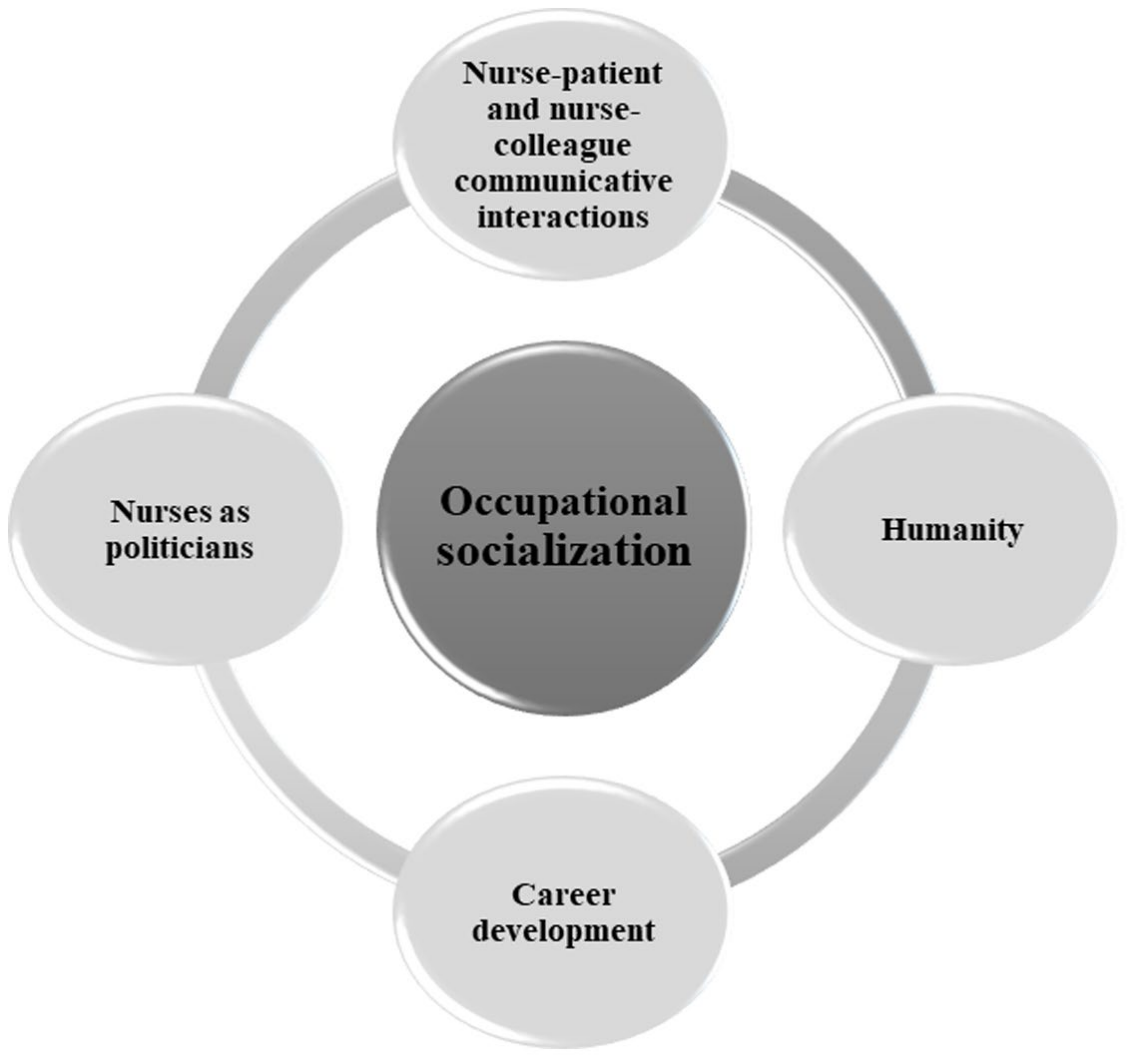
Education necessary to improve nursing governance.

Qualitative data were analyzed via thematic analysis. The thematic analysis method outlined by Braun and Clarke (17) was utilized, encompassing the subsequent steps: gaining familiarity with the data, formulating initial codes, identifying themes, reviewing the themes, defining and labeling the themes, and writing the report. The author read through the data initially to gain familiarity and to assign codes. The field notes were useful to recall the air of each interview. A total of 18 codes were developed and checked for duplication or multiple codes expressing the same concept. The codes were organized into sub-themes and themes that the researchers felt represented the participants’ responses. These final themes and sub-themes are illustrated in Figure 1. In the final stage of analysis, quotes that the researchers felt were accurate examples highlighting the themes were selected from the data. For the general characteristics of the participants, descriptive statistics using Microsoft 365 Excel were calculated.

### 2.5. Rigor and trustworthiness

The transcribed data were repeatedly reviewed to improve validity, transparency, and quality until no new concepts were generated. Since the author conducted the data analysis, the finalized and main themes were emailed to the participants to ensure their responses were adequately reflected.

### 2.6. Ethical consideration

Ethical approval was obtained from the author’s institutional review board before data collection. Informed consent was obtained from all participants. All data and quotes were pseudonymized. This study adheres to the Declaration of Helsinki in all methodological aspects.

## 3. Results

A total of 14 participants were interviewed regarding nursing educational content to improve governance in nursing. All participants identified as women shared common characteristics: they were all over 38 years of age with the mean (SD) value of 44.79 (4.41) and they held a minimum of a master’s degree as their highest level of education. Their average career years as a nurse manager were 7–8 years with the mean (SD) value of 7.46 (5.95). Data analysis revealed one main theme and four sub-themes. An overview of the themes is illustrated in Figure 1.

### 3.1. Occupational socialization

The main theme, “occupational socialization,” appeared in the analysis of education needed in undergraduate programs to improve nursing governance. The sub-themes of communication with patients and colleagues, humanity, career development, and political participation were derived to describe the main theme.

#### 3.1.1. Nurse–patient and nurse-colleague communicative interactions

Education on communication with patients and colleagues was considered necessary to enhance nursing governance. Such communication meant education on technical communication such as etiquette and Situation-Background-Assessment-Recommendation (SBAR), which should be performed in relationships with colleagues in a healthcare organization. Particularly, such communication skills are useful when presenting their opinions in committees. Moreover, to enhance the professionalism of nursing services based on patients’ needs, the necessity of experiencing communication with various people was suggested.

Communication is very important, and interpersonal relationships are in consecutive order; however, nowadays, nurses do not communicate. They were also unaware of courtesy (etiquette) in communication with physicians, nurses, and senior nurses. Courtesy is required during communication in a healthcare organization (Participant 10).

We provided nurses with SBAR training. (···) We explained to new nurses how to communicate and perform SBAR when notifying doctors from the first orientation training, but they still do not…proficiently. These skills are necessary when nurses speak in committees or conferences (Participant 8).

I had many opportunities in nursing school to communicate with friends majoring in other subjects. I think talking and listening to as many people as possible is important. It helps nurses when dealing with patients’ needs in a hospital. It helps nurses understand what the patient wants quickl (Participant 9).

#### 3.1.2. Humanity

Nursing is a patient-centered care service. Participants stated that attention to the human aspect of nursing is needed to improve nursing governance. Additionally, as such attention is linked to reflection and nursing ethics, the need for education to cultivate humanistic knowledge was mentioned.

When caring for patients, we need to understand humans. Even though I am a nurse providing nursing services to psychiatric patients, there are times when I wonder if I have an interest in patients or understand human beings when I cannot understand a patient’s behavior (Participant 11).

Nurses can be unskilled in functional tasks, but we focus on whether the nurse paid attention to patients with humanistic interest. Nurses who are interested in themselves tend to pay significant attention to patients. Nurses should be able to reflect on themselves and look inside their minds, but they do not know how to do that (Participant 12).

#### 3.1.3. Career development

Participants suggested that education on career development as a nurse is necessary. As such, they mentioned education about clinical careers and human resources in healthcare organizations. Through education, nurses could have improved insights into healthcare staff management. Also, they could be convinced of their potential and strive to develop their career.

I think that I have to learn about human resources (HR). I can develop through such education. If more intensive training on the development and HR part is provided, I would better understand the management of healthcare personnel and ensure that I am a nurse who can grow as an excellent nurse (Participant 8).

We need an education that can teach nurses in advance about activities that can help them develop nursing careers in the wards so that they feel like they can work as a nurse for a long time (Participant 5).

#### 3.1.4. Nurses as politicians

Participants emphasized the need for education on political participation. They reflected that they were not interested in such education. However, while considering ways to improve nurses’ governance, they reflected on nurses who had not given this much consideration to politics and urged them to increase their knowledge and interest. As nurses’ involvement in enacting the Nursing Act is needed, nurses’ participation in decision-making is needed to improve nursing governance.

Last year, I attended lectures on politics while preparing for the enactment of the Nursing Act. Ultimately, it is about political involvement, like governance is about participation. But we nurses did not give this much consideration. I think many nurses are unaware of the department focused on nursing politics newly established under the Ministry of Health and Welfare (Participant 7).

[To enact the Nursing Act, nurses] have to put pressure on and negotiate with politicians such as members of the National Assembly. There is a need to increase the number of nurse activists and nurse politicians. Currently, there are few nurse politicians to represent the voices of nurses. Thus, nursing students need to become interested in politics in nursing school through education (Participant 13).

## 4. Discussion

This study aimed to identify undergraduate nursing programs’ educational content to improve nurses’ governance in South Korea. Nurse managers suggested the necessity of education for occupational socialization, highlighting the need for education on nurse–patient and nurse-colleague communicative interactions, humanity, career development, and political involvement.

The main theme identified in this study was “occupational socialization.” Occupational socialization embraces the entire process of novices in an organization by adjusting themselves to the organization through learning its professional codes and values, culture, performing an expected job, and obtaining the skills necessary (18). A related theory in the field of physical education is the occupational socialization theory. Its framework comprises a time-oriented continuum: acculturation, professional socialization, and organizational socialization (19). The educations on communication, humanity, career development, and political involvement, which were this study’s sub-themes, align with the suggested framework. Particularly, education on career development and political involvement can be viewed as related to professional and organizational socialization.

The first specific form of education required in nursing governance is nurse–patient and nurse-colleague communicative interactions. Guttman et al. (20) outlined that communication is one of the strategies to improve patient safety, but it remains an adaptive challenge to overcome in the healthcare sector. Appropriate communication can enhance nurses’ work lives by increasing their satisfaction with professional communication and reducing Thas among colleagues (21). Moreland and Apker (22) also identified that communicative responses reduce conflict and stress among nurses and highlighted that communication training should begin in undergraduate nursing program to provide experiential learning techniques to create a culture of respect. Professional communication is likely related to participation in committees, a subdomain of governance. When nurses are involved in committees to make decisions on policies and nursing practice, they should present their opinions professionally. In South Korea, a study analyzing 48 out of 73 undergraduate nursing curriculums in 2009 revealed that 81.2% recognized the importance of communication and already had communication as a major course (23). This important communication education is covered in a single course with 2 credits in the first or second year of the four-year curriculum, and the lack of communication experience has been highlighted (23, 24). Thus, expanding or increasing credits for communication course(s) in undergraduate nursing programs in South Korea should be considered.

Additionally, our findings revealed the need for communication education, particularly regarding courtesy during communication with colleagues. It implies the need for organizational communication experience with colleagues as a member of the healthcare organization. Organizational communication involves complex interpersonal relationships (25). Thus, many communication experiences with various occupations in healthcare organizations is encouraged for undergraduate nursing programs.

Humanity is the second aspect of education needed for nursing governance. In particular, the participants indicated that nurses need to have an attitude to try to know and understand patients from a humanistic perspective. Patients are an important component of the metaparadigm of nursing (26, 27). Additionally, the concept of person-centered care has been introduced, and the American Association of Colleges of Nursing (28) defines it as holistic, personalized care delivered with respect and compassion, which guides nursing practice regardless of specialty and emphasizes the importance of liberal arts, such as humanities, for professional nursing education. Byma and Lycette (29) found that nursing students recognized the benefits of humanities-based activities, such as emotional development, communication, and new insights into best nursing practices. The current study derived a similar finding that interest in and understanding patients (human beings) are linked to reflection as a nurse. In other words, reflection on the nursing service spent on patient care is expected to positively impact the improvement of the control over practice areas in nursing governance. A total of 8 credits (4 courses) of the total graduation credits (130–140 credits) in nursing undergraduate courses in South Korea are awarded for humanity education (30). Nevertheless, the fact that such education was mentioned as necessary to improve nursing governance suggests the need to examine the effectiveness of such courses in the current nursing undergraduate curriculum.

Education on career development is the third type needed for nursing governance. Participants mentioned the need for education on HR, which is conducive to careers and can provide growth potential for nurses in health organizations. Moreover, it could affect nurses’ understanding of the management of healthcare personnel. Notably, considering the results of a study (31) targeting Generation Y nurses born in the 1980s, they no longer want to be leaders and want to work as general nurses; this highlights the fact that education on career development is necessary. In South Korea, the concept of the career ladder system and its example cases have been introduced into the undergraduate nursing curriculum. However, this does not seem sufficient. Kalbfleisch and Burwell (32) suggested career-specific education for nursing practitioners in Canada. Thus, the nursing career-specific development process in tertiary and general healthcare organizations nationwide should be analyzed to develop educational content. The content finalized with experts can be utilized in undergraduate nursing programs and healthcare organizations; thereby, nursing students are likely to have an improved understanding of career development and control over personnel in nursing governance.

Political involvement was the fourth type of education required for professional governance in nursing. Nurses’ political participation is essential for developing effective health programs and global health promotion (33). A recent study reported that students of nursing majors’ political interest and participation are higher than those of other majors, but their political efficacy is low (34). Political efficacy refers to the subjective emotional judgment of how much a political actor can change the political environment with their efforts (35). It can be interpreted as confidence in politics. Even after becoming a nurse, owing to excessive stress and shift work related to the life and safety of the patient, the knowledge and confidence related to political participation are low compared to other medical-related occupations (36). Thus, nurses do not participate in healthcare policy decisions (37). Korean nurses have tried enacting the Nursing Act to specify the scope of their work according to the law. However, the fact that the enactment of the Nursing Act has still not been established implies the need to improve the political capacity of Korean nurses.

Regarding political involvement education, considering the subdomains of governance, namely control over personnel, control over practice, influence over resources, access to information, participation in committee structure, goal setting, and conflict resolution (1), governance is the authority given to nurses. With the concept of making good use of authority, governance in nursing can be viewed as a political activity in hospitals in a broad sense. Just as citizens exercise citizenship, nurses in hospitals should exercise the authority and opportunities given to them to raise their voices and participate in decision-making related to nursing practices (38). For this purpose, politics as educational content is needed in undergraduate nursing programs so that they can understand the definition of politics and the legislative process (39) in advance.

Since only a few studies about nursing governance from nurse managers’ perspectives have been conducted, this study’s findings are considered important but limited in scope. The data were collected from the participants whose institutions were general or tertiary hospitals, therefore this study’s findings may not be generalizable to nurse managers’ perceptions in primary healthcare institutions. Additionally, the data were analyzed by a single author. Thus, the author ensured that the results of this study contained the participants’ intention through the feedback process with them.

Lastly, the interviews were conducted individually. Focus group interviews with nurse managers could provide rich data regarding nursing governance, although data saturation was achieved in this study.

## 5. Conclusion

This study aimed to identify nursing education methods for improving nurses’ governance by targeting head nurses. This study found that necessary education was mainly related to organizational socialization. Specifically, education on nurse–patient and nurse-colleague communicative interactions, humanity, career development, and nurses as politicians were identified. First, regarding communication and etiquette between colleagues and patients, it is necessary to provide an educational environment in which nursing students can experience communication with various occupational groups engaged in medical institutions. Second, for the education on humanity, which includes an understanding of patients and is connected to reflection as a nurse and nursing ethics, it is necessary to check the effectiveness of such courses currently organized in nursing educational institutions. It may positively impact the improvement of the control over practice area through reflection on patient nursing work. Third, education on career development suggests the need for career-specific education to ensure the growth potential of a nurse within the organization. Thus, the control over personnel in nursing governance is expected to be enhanced. Fourth, education on political involvement is required for nurses’ political participation. Governance can be viewed similarly as political activities within medical institutions. Therefore, it is necessary to prepare educational programs on “Nursing and Politics” in nursing educational institutions. Through education on communication, humanity, career development, and politics, the occupational socialization of nurses should occur gradually, thereby improving nursing governance. This study is meaningful in that it suggests vital educational content and the direction of education in undergraduate nursing programs to improve the governance of nurses in a situation where Korean nurses are confronting challenges moving toward shared governance in nursing (5). Further studies should examine the effects of these four themes on nursing governance in South Korea using surveys.

## Data availability statement

The original contributions presented in the study are included in the article/supplementary material, further inquiries can be directed to the corresponding author.

## Ethics statement

The studies involving humans were approved by Woosuk University Institutional Review Board. The studies were conducted in accordance with the local legislation and institutional requirements. The participants provided their written informed consent to participate in this study.

## Author contributions

SC: Conceptualization, Data curation, Formal analysis, Investigation, Methodology, Validation, Visualization, Writing – original draft.

## Funding

The author declares that this study received funding from the Korea government (MSIT) (No. NRF-2019R1G1A1008098) and the Soonchunhyang University Research Fund. The funder was not involved in the study design, collection, analysis, interpretation of data, the writing of this article, or the decision to submit it for publication.

## Conflict of interest

The author declares that the research was conducted without any commercial or financial relationships that could be construed as a potential conflict of interest.

## Publisher’s note

All claims expressed in this article are solely those of the authors and do not necessarily represent those of their affiliated organizations, or those of the publisher, the editors and the reviewers. Any product that may be evaluated in this article, or claim that may be made by its manufacturer, is not guaranteed or endorsed by the publisher.
